# ERGA-BGE reference genome of the Azores Bullfinch - *Pyrrhula murina* Godman, 1866: an IUCN Vulnerable Species endemic to a single island in the Azores Archipelago (Portugal)

**DOI:** 10.12688/openreseurope.20666.3

**Published:** 2026-03-11

**Authors:** Ricardo Jorge Lopes, Astrid Böhne, Thomas Marcussen, Rebekah A. Oomen, Torsten Hugo Struck, Laura Aguilera, Marta Gut, Francisco Câmara Ferreira, Fernando Cruz, Jèssica Gómez-Garrido, Tyler S. Alioto, Rita Monteiro

**Affiliations:** 1 CE3C, Center for Ecology, Evolution and Environmental Change & CHANGE, Departamento de Biologia Animal, Faculdade de Ciências, Universidade de Lisboa, Lisboa, Portugal; 2 MUHNAC, Museu Nacional de História Natural e da Ciência, Universidade de Lisboa, Rua da Escola Politécnica, Lisboa, Portugal; 3 Leibniz Institute for the Analysis of Biodiversity Change, Museum Koenig Bonn, Bonn, 53113, Germany; 4 Natural History Museum, University of Oslo, Oslo, Norway; 5 Centre for Ecological & Evolutionary Synthesis, University of Oslo, Oslo, Norway; 6 Department of Biological Sciences, University of New Brunswick Saint John, Saint John, Canada; 7 Tjärnö Marine Laboratory, University of Gothenburg, Gothenburg, Sweden; 8 Centre for Coastal Research, University of Agder, Kristiansand, Norway; 9 Centro Nacional de Análisis Genómico (CNAG), Barcelona, Spain; 10 Universitat de Barcelona (UB), Barcelona, Spain

**Keywords:** Pyrrhula murina, Fringillidae family, Azores Bullfinch, genome assembly, European Reference Genome Atlas, Biodiversity Genomics Europe, Earth Biogenome Project

## Abstract

*The reference genome of Pyrrhula murina*’s reference genome will substantially enhance the current monitoring of genetic diversity and population viability and will allow us to understand the effective population size trends throughout time and recent bottlenecks and population expansions. A total of 42 contiguous chromosomal pseudomolecules were assembled from the genome sequence, using ONT, HI-C and Illumina data. This chromosome-level assembly encompasses 1.2 Gb, composed of 65 contigs and 60 scaffolds, with contig and scaffold N50 values of 65 Mb and 76 Mb, respectively.

## Introduction

**Figure 1.**
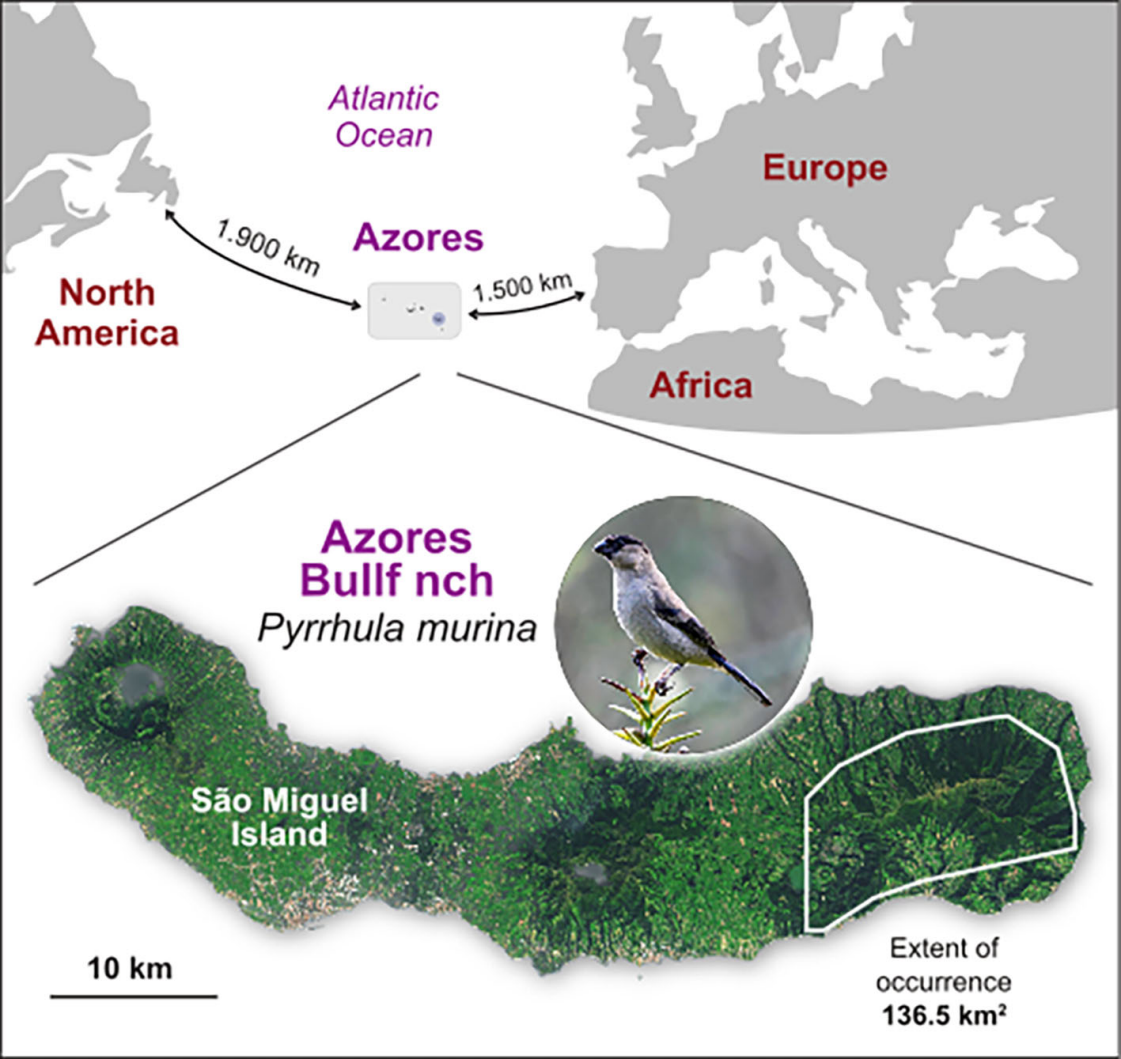
Spatial location of the Azores archipelago and the distribution range of the Azores Bullfinch ( *Pyrrhula murina*) in the east part of São Miguel Island. Also shown the latest estimates of the extent of occurrence, from Costa et al. (2023).

The Azores Bullfinch, *Pyrrhula murina* Godman, 1866, is restricted to a small area that includes native laurel forest, in the east of the largest island (São Miguel) of the Azores archipelago ( Figure 1). It is thought to have diverged from its nearest sister species more than 1 MYA ( Töpfer *et al.*, 2011).

By the late 19th/early 20th century, the Azores Bullfinch was already very rare and restricted to the higher mountain valleys in the east of São Miguel island ( Aubrecht, 2000). It was rediscovered only in the last century, and numbers have increased as a result of conservation efforts to protect this species and its main habitat. Its current population size is estimated at around 1000 individuals ( Ceia *et al.*, 2011; Costa *et al.*, 2023; Gil *et al.*, 2016), and its conservation status has been down-listed from “Critically Endangered” to “Vulnerable” on the IUCN Red List, as the population size is considered to be stable ( BirdLife International, 2021).

It is one of the few endemic bird species that is strongly connected to the last remnants of the Laurel Forest in the Azores, providing key ecological services for the sustainability of these habitats.

A high-quality reference genome will allow us to enhance our knowledge on the long-term viability of this small population, informing us about the impact of the multiple bottlenecks on its genetic diversity and also on the current and future evolution of this population. Of particular importance for the viability of the species, is the fact that *Pyrrhula murina*, along with his sister species, the Eurasian Bullfinch, *Pyrrhula pyrrhula*, have relatively small and possibly neotenous sperm, an ancestral trait that evolved before the two taxa diverged ( Lifjeld *et al.*, 2013).

The generation of this reference resource was coordinated by the European Reference Genome Atlas (ERGA) initiative’s Biodiversity Genomics Europe (BGE) project, supporting ERGA’s aims of promoting transnational cooperation to promote advances in the application of genomics technologies to protect and restore biodiversity ( Mazzoni *et al.*, 2023).

## Materials & methods

ERGA’s sequencing strategy includes Oxford Nanopore Technology (ONT) and/or Pacific Biosciences (PacBio) for long-read sequencing, along with Hi-C sequencing for chromosomal architecture, Illumina Paired-End (PE) for k-mer profiling and polishing (i.e. recommended for ONT-only assemblies), to facilitate genome assembly, as well as Illumina RNA sequencing for transcriptomic profiling and annotation.

### Sample and sampling information

On November 27, 2023, a female adult (determined based on genetic sexing) of *Pyrrhula murina* was sampled and identified by Ricardo Jorge Lopes. The specimen was caught using mist-nets in a woodland area in Salto do Cavalo, São Miguel island, in the Azores archipelago, Portugal.

This species is unmistakable, being the sole representative of this Genus in the Azores Archipelago.

Sampling was performed under permit 119/2023/DRAAC, from the Regional Directorate for the Environment and Climate Action, of the Azores Government, and under the Internationally Recognized Compliance certificate CCIR-RAA/2023/65. The specimen’s blood samples were snap-frozen immediately after harvesting and stored in liquid nitrogen until DNA extraction.

### Vouchering information

An electronic voucher image of the sequenced individual is available from ERGA’s EBI BioImageArchive dataset www.ebi.ac.uk/biostudies/bioimages/studies/S-BIAD1012?query=ERGA under accession ID SAMEA115966740.

A frozen reference blood sample from the sequenced individual was deposited at MUHNAC (National Museum of Natural History and Science of the University of Lisbon), under the voucher ID C67972_Blood_05.

### Genetic information

The estimated genome size, based on ancestral taxa, is 1.33 Gb, while the estimation based on reads kmer profiling using GenomeScope2 (kmer length = 21) is 1.08 Gb. This is a diploid genome with a haploid number of 39 chromosomes (2 *n* = 78), including Z and W sex chromosomes in females, based on ancestral data (no direct estimates are available. All information for this species was retrieved from Genomes on a Tree ( Challis *et al.*, 2023).

### DNA/RNA processing

DNA was extracted from blood using the Blood & Cell Culture DNA Midi Kit (Qiagen) following the manufacturer’s instructions. DNA quantification was performed using a Qubit dsDNA BR Assay Kit (Thermo Fisher Scientific), and DNA integrity was assessed using a Genomic DNA 165 Kb Kit (Agilent) on the Femto Pulse system (Agilent). The DNA was stored at +4 °C until sequenced.

RNA was extracted from blood using an RNeasy Mini Kit (Qiagen) according to the manufacturer’s instructions. RNA quantification was performed using the Qubit RNA BR kit, and RNA integrity was assessed using a Bioanalyzer 2100 system (Agilent) RNA 6000 Nano Kit (Agilent). The RNA was stored at -80 °C until sequenced.

### Library preparation and sequencing

For long-read whole genome sequencing, a library was prepared using the SQK-LSK114 Kit (Oxford Nanopore Technologies, ONT), which was then sequenced using a R10.4.1 flow cell on a PromethION 24 A Series instrument (ONT). Basecalling was performed using Dorado v7.4.14 (Oxford nanopore Technologies) with the super accurate (SUP) basecalling model v4.3.0 at 400 bps, integrated within MinKNOW v24.06.15. A short-read whole-genome sequencing library was prepared using the KAPA Hyper Prep Kit (Roche).

A Hi-C library was prepared from blood using the Dovetail Omni-C Kit (Cantata Bio), followed by the KAPA Hyper Prep Kit for Illumina sequencing (Roche).

The RNA library was prepared using the KAPA mRNA Hyper prep kit (Roche). All short-read libraries were sequenced on a NovaSeq 6000 instrument (2x150 bp, Illumina).

In total, 100x Oxford Nanopore, 99x Illumina WGS shotgun, and 79x Hi-C data were sequenced to generate the assembly. In addition, 91 million reads of Illumina RNA-seq were sequenced to facilitate future annotation efforts.

### Genome assembly methods

The genome was assembled using the CNAG CLAWS pipeline v2.3.0 ( Gomez-Garrido, 2024). Briefly, ONT reads were preprocessed for quality and length using Filtlong v0.2.1 (--min_length 1000 --min_mean_q 97 -t 80000000000) , and initial contigs were directly assembled from the filtered ONT data using Hifiasm v0.24.0 with the –ont option and Hi-C phasing, followed by scaffolding with YaHS v1.2a using the Hi-C data. Assembled scaffolds were curated via manual inspection using Pretext v0.2.5 with the Rapid Curation Toolkit ( https://gitlab.com/wtsi-grit/rapid-curation ) to remove any false joins and incorporate any sequences not automatically scaffolded into their respective locations in the chromosomal pseudomolecules (or super-scaffolds). The blobtoolkit nextflow pipeline v0.6.0 ( https://pipelines.tol.sanger.ac.uk/blobtoolkit/0.6.0/usage) confirmed the absence of contaminants. The sex chromosomes were identified by sequencing coverage. Assignment of Z and W chromosomes was according to conservation of length: bPyrMur chrZ (OZ238997.1: 86793913 bp) vs. bGalGal chrZ (CM028522.1: 86044486 bp). Whole genome alignment against GRCg7b (GCA_016699485.1) confirmed these assignments. Finally, the mitochondrial genome (OZ239036.1) was assembled as a single circular contig of 16,836 bp using the FOAM pipeline v0.5 ( https://github.com/cnag-aat/FOAM) and included in the released assembly (GCA_965183895.1). K-mer counts for estimating genome size, QV and k-mer completeness were performed on the combined set of Illumina WGS and filtered ONT reads. Summary analysis of the released assembly was performed using the ERGA-BGE Genome Report ASM Galaxy workflow ( De Panis, 2024).

## Results

### Genome assembly

**Figure 2.**
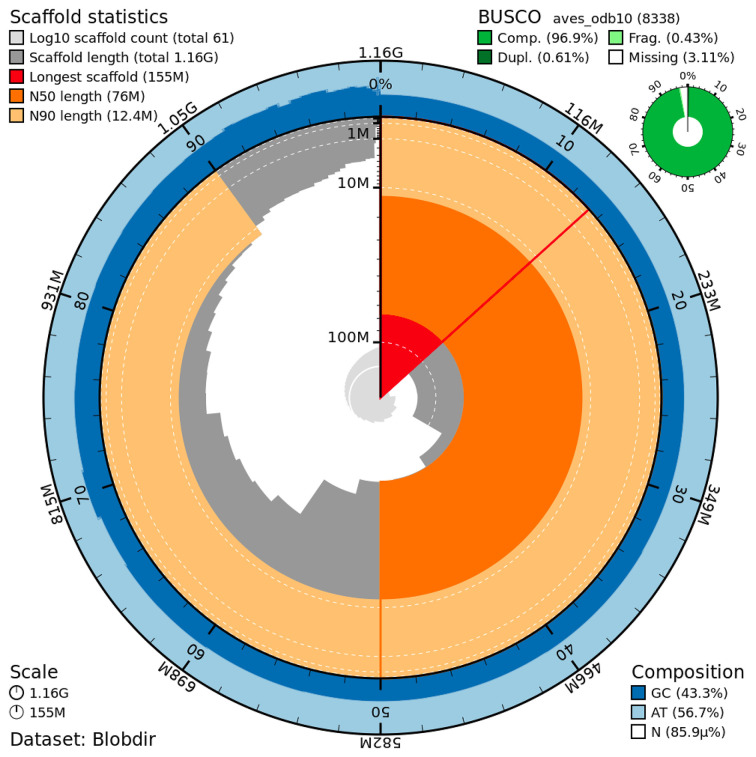
Snail plot summary of assembly statistics. The main plot is divided into 1,000 size-ordered bins around the circumference, with each bin representing 0.1% of the 1,163,884,374bp assembly, including the mitochondrial genome. The distribution of sequence lengths is shown in dark grey, with the plot radius scaled to the longest sequence present in the assembly (155 Mb bp, shown in red). Orange and pale-orange arcs show the scaffold N50 and N90 sequence lengths (76,036,967 and 12,418,366 bp), respectively. The pale grey spiral shows the cumulative sequence count on a log-scale, with white scale lines showing successive orders of magnitude. The blue and pale-blue area around the outside of the plot shows the distribution of GC, AT, and N percentages in the same bins as the inner plot. A summary of complete, fragmented, duplicated, and missing BUSCO genes found in the assembled genome from the avian database (odb10) is shown on the top right.

**Figure 3.**
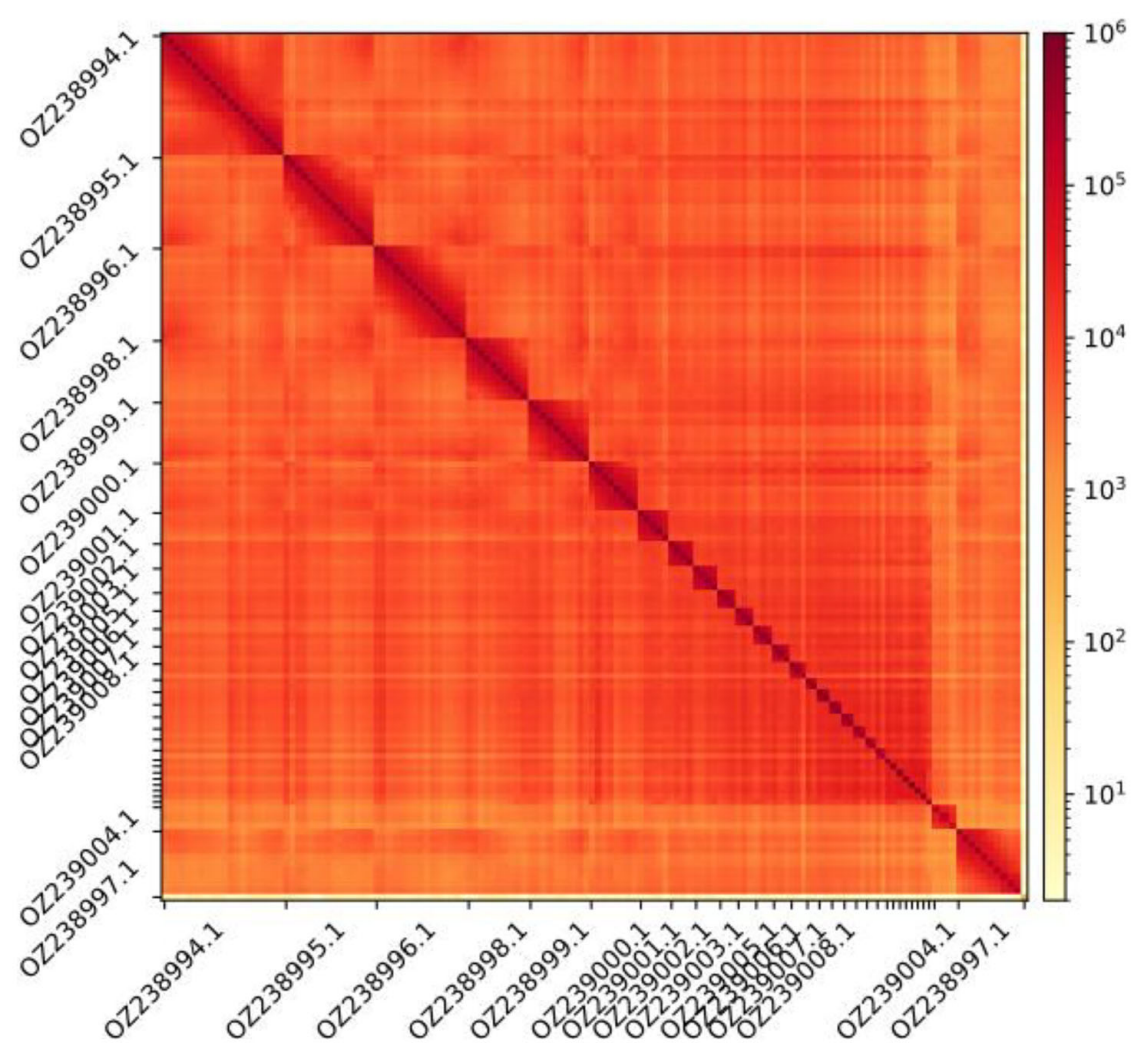
Hi-C contact map showing spatial interactions between regions of the genome. The diagonal corresponds to intra-chromosomal contacts, depicting chromosome boundaries. The frequency of contacts is shown on a logarithmic heatmap scale. Hi-C matrix bins were merged into a 150 kb bin size for plotting. Due to space constraints on the axes, only the GenBank names of the 13th largest autosomes and the sex chromosomes are shown.

The genome assembly has a total length of 1,163,884,374 bp in 61 scaffolds, including 40 autosomes, the Z and W chromosomes, and the mitogenome ( Figure 2 and Figure 3), with a GC content of 43.29%. It features a contig N50 of 64,819,875 bp (L50=6) and a scaffold N50 of 76,036,967 bp (L50=6). There are 5 gaps, totaling 1,000 kb in cumulative size. The ten largest autosomes as well as the Z and W scaffolds are longer than 28 Mb and the rest (30 autosomes) are shorter than 23 Mb, the shortest being about 3.5 Mb. The single-copy gene content analysis using the aves_odb10 database with BUSCO v5.5.0 ( Manni *et al.*, 2021) resulted in 96.9% completeness (96.3% single and 0.6% duplicated). 98.6% of reads k-mers were present in the assembly, and the assembly has a base accuracy Quality Value (QV) of 52.6 as calculated by Merqury v1.3 ( Rhie *et al.*, 2020).

## Author contributions

RJL coordinated the project, collected the species, identified the species, sampled and preserved biological material and provided metadata, RM, TM, RAO, THS, and AsB provided sampling and metadata support and management, LA and MG extracted DNA, prepared libraries, and performed sequencing, FCF, JGG and FC performed genome assembly and curation under the supervision of TA, RM generated the analysis and report. All authors contributed to the writing, review, and editing of this genome note and read and approved the final version.

## Data Availability

*Pyrrhula murina* and the related genomic study were assigned to Tree of Life ID (ToLID) ‘bPyrMur1’, and all sample, sequence, and assembly information are available under the umbrella BioProject PRJEB86373 from https://www.ebi.ac.uk/ena/browser/view/PRJEB86373. The sample information is available at the following BioSample accessions: SAMEA115966741 and SAMEA115966742. The genome assembly is accessible from ENA under accession number GCA_965183895. Sequencing data produced as part of this project are available from ENA at the following accessions: ERX14064180, ERX14064181, and ERX14064182. Documentation related to the genome assembly and curation can be found in the ERGA Assembly Report (EAR) document available at https://github.com/ERGA-consortium/EARs/tree/main/Assembly_Reports/Pyrrhula_murina/bPyrMur1. The mitochondrial genome was assembled into a single circular contig using the FOAM pipeline v0.5 ( https://github.com/cnag-aat/FOAM). Further details and data about the project are hosted on the ERGA portal at https://portal.erga-biodiversity.eu/data_portal/928672.
