# Supplementary material for: ERGA-BGE reference genome of the Azores Bullfinch - Pyrrhula murina Godman, 1866: an IUCN Vulnerable Species endemic to a single island in the Azores Archipelago (Portugal)
Source: Open Res Eur. 2026 Mar 11;5:210. Originally published 2025 Aug 1. [Version 3] doi: 10.12688/openreseurope.20666.3 (PMC12982980; doi:10.12688/openreseurope.20666.3)
Supplement: Peer review history

# Reviewer report: Reviewer response for version 3

**DOI:** 10.21956/openreseurope.25295.r71045

**Published:** 2026-04-15

**Authors:** Annabel Whibley

**Affiliations:** 1 Grapevine Improvement, Bragato Research Institute, Lincoln, New Zealand; 2 University of Auckland, Auckland, New Zealand

No additional comments

Are sufficient details of methods and materials provided to allow replication by others?

Partly

Is the rationale for creating the dataset(s) clearly described?

Yes

Are the datasets clearly presented in a useable and accessible format?

Yes

Are the protocols appropriate and is the work technically sound?

Yes

Reviewer Expertise:

Genomics, Bioinformatics

I confirm that I have read this submission and believe that I have an appropriate level of expertise to confirm that it is of an acceptable scientific standard.

# Reviewer report: Reviewer response for version 3

**DOI:** 10.21956/openreseurope.25295.r71044

**Published:** 2026-03-13

**Authors:** Luke W Silver

**Affiliations:** 1 University of Sydney, Sydney, Australia

No further comments

Are sufficient details of methods and materials provided to allow replication by others?

Partly

Is the rationale for creating the dataset(s) clearly described?

Yes

Are the datasets clearly presented in a useable and accessible format?

Yes

Are the protocols appropriate and is the work technically sound?

Yes

Reviewer Expertise:

Genome assembly, genetic diversity

I confirm that I have read this submission and believe that I have an appropriate level of expertise to confirm that it is of an acceptable scientific standard.

# Reviewer report: Reviewer response for version 2

**DOI:** 10.21956/openreseurope.24922.r69658

**Published:** 2026-03-12

**Authors:** Madeline Chase

**Affiliations:** 1 Swiss Ornithological Institute, Sempach, Switzerland

The authors generate a high quality, chromosome scale genome assembly for the Azores Bullfinch. This is a vulnerable species with a restricted range and low population size, and the reference genome may aid conservation efforts. 

 The authors generally provide sufficient details on their methods; however, citations are missing for several of the software used in the genome assembly section that should be provided. Hifiasm and YaHS for instance should include citations. 

 One other note is that in the introduction, this sentence needs revision: "Of particular importance for the viability of the species, is the fact that Pyrrhula murina, along with his sister species," 

 " *Pyrrhula murina" *should be in italics and "his" should be their or its.

Are sufficient details of methods and materials provided to allow replication by others?

Partly

Is the rationale for creating the dataset(s) clearly described?

Yes

Are the datasets clearly presented in a useable and accessible format?

Yes

Are the protocols appropriate and is the work technically sound?

Yes

Reviewer Expertise:

Population and speciation genomics

I confirm that I have read this submission and believe that I have an appropriate level of expertise to confirm that it is of an acceptable scientific standard, however I have significant reservations, as outlined above.

# Reviewer report: Reviewer response for version 2

**DOI:** 10.21956/openreseurope.24922.r69516

**Published:** 2026-02-27

**Authors:** Annabel Whibley

**Affiliations:** 1 Grapevine Improvement, Bragato Research Institute, Lincoln, New Zealand; 2 University of Auckland, Auckland, New Zealand

The authors have satisfactorily addressed my earlier concerns and I express my thanks for their engagement and efforts.

 Minor comments:

 Please note that " *Pyrrhula murina" *needs italicizing in the 4th paragraph of the introduction. 

 I would also like to see the basecalling model and dorado version specified for the ONT sequencing (my apologies for not flagging this earlier).

Are sufficient details of methods and materials provided to allow replication by others?

Partly

Is the rationale for creating the dataset(s) clearly described?

Yes

Are the datasets clearly presented in a useable and accessible format?

Yes

Are the protocols appropriate and is the work technically sound?

Yes

Reviewer Expertise:

Genomics, Bioinformatics

I confirm that I have read this submission and believe that I have an appropriate level of expertise to confirm that it is of an acceptable scientific standard.

# Response

**Published:** 2026-03-05

**Authors:** Rita Monteiro

We thank the reviewer for these suggestions. 1.Corrected  2. Added to the main text: Basecalling was performed using Dorado v7.4.14 (Oxford nanopore Technologies) with the super accurate (SUP) basecalling model v4.3.0 at 400 bps, integrated within MinKNOW v24.06.15.

# Reviewer report: Reviewer response for version 2

**DOI:** 10.21956/openreseurope.24922.r69517

**Published:** 2026-02-27

**Authors:** Luke W Silver

**Affiliations:** 1 University of Sydney, Sydney, Australia

The authors have addressed all of my concerns and the additional methods included have improved the manuscripts. One final comment is that the authors have undertaken repeatmasking of the assembly, could they please include the methods and the results (in the form of a table of repeat types) in the manuscript

 Competing Interests: No competing interests were discl

Are sufficient details of methods and materials provided to allow replication by others?

Partly

Is the rationale for creating the dataset(s) clearly described?

Yes

Are the datasets clearly presented in a useable and accessible format?

Yes

Are the protocols appropriate and is the work technically sound?

Yes

Reviewer Expertise:

Genome assembly, genetic diversity

I confirm that I have read this submission and believe that I have an appropriate level of expertise to confirm that it is of an acceptable scientific standard, however I have significant reservations, as outlined above.

# Response

**Published:** 2026-03-05

**Authors:** Rita Monteiro

We thank the reviewer for this suggestion. However, ERGA-BGE genome reports are specifically scoped to present the assembly and its core metrics, rather than provide detailed genomic analyses. Additionally, including a full repeat breakdown could conflict with more in-depth work being carried out by the sample provider for future publications. We have therefore respectfully maintained the current level of detail in this section.

# Reviewer report: Reviewer response for version 1

**DOI:** 10.21956/openreseurope.22356.r57666

**Published:** 2025-09-12

**Authors:** Annabel Whibley

**Affiliations:** 1 Grapevine Improvement, Bragato Research Institute, Lincoln, New Zealand; 2 University of Auckland, Auckland, New Zealand

Lopes and colleagues describe the generation of a reference genome assembly for the Azores Bullfinch, *Pyrrhula murina*, a species classified as Vulnerable by the IUCN. The quality of the genome is high, with >96% BUSCO completeness and a base-level accuracy of 52.6QV. The assembly has been scaffolded into chromosomal pseudomolecules, although no comment is made on the discrepancy between the 42 pseudochromosomes recovered and the expected 2n=78 nor to whether any of the scaffolds/contigs remain unlinked to chromosomes.

 The assembly and supporting data have been publicly released and the accession links are active. The authors describe obtaining RNA sequencing data but an annotation is not provided here.

 While the manuscript is generally well-structured and is close to the Data Note template used by the Darwin Tree of Life consortium, there are notable gaps in the reporting of methods and important metadata details. For example, the methods section presents a genome size estimate from kmer analysis without noting how this method was obtained (GenomeScope2 with a kmer length of X?). The versions of software used are not always reported and key parameters are sometimes omitted. What are the parameters used to filter the sequencing reads? What are the properties (beyond coverage) of the ONT libraries? The flow cell type (R10.4.1) should probably be reported for the ONT sequencing along with the library type.  Methods reporting manual curation would typically also give an indication of the number and type of interventions.

 Some of the language is a little informal. For example, the opening of the abstract with a possessive rather than “The reference genome of…” is unusual. And “This kind of high-quality…” in the introduction is vague.

 This sentence needs revision: “Of particular importance for its viability, is the fact that, along with his sister species, the Eurasian Bullfinch, *Pyrrhula pyrrhula*, they have relatively small and possibly neotenous sperm, an ancestral trait that evolved before the two taxa diverged (Lifjeld et al., 2013).” I would suggest :  Of particular importance for **the viability of the species**, is the fact that ***Pyrrhula murina*** **,** along with **a** sister species, the Eurasian Bullfinch, *Pyrrhula pyrrhula*, have relatively small and possibly neotenous sperm, an ancestral trait that evolved before the two taxa diverged (Lifjeld et al., 2013).”

 The introduction of the methods describes RNA sequencing data without noting the sequencing type and states that it is used to facilitate genome assembly and annotation, with scant methodological evidence that the RNA dataset has informed the assembly generation.

 The latitude and longitude co-ordinates for the collection site should be included.

Are sufficient details of methods and materials provided to allow replication by others?

Partly

Is the rationale for creating the dataset(s) clearly described?

Yes

Are the datasets clearly presented in a useable and accessible format?

Yes

Are the protocols appropriate and is the work technically sound?

Yes

Reviewer Expertise:

Genomics, Bioinformatics

I confirm that I have read this submission and believe that I have an appropriate level of expertise to confirm that it is of an acceptable scientific standard, however I have significant reservations, as outlined above.

# Response

**Published:** 2026-01-29

**Authors:** Rita Monteiro

We thank the reviewer for their comments. We have addressed all of them as following:

1.  The discrepancy between the previously reported of  39 pseudochromosomes (2n=78)  and the 42 pseudochromosomes recovered in our assembly can be explained by differences in how the chromosome number was inferred. The number 2n=78 pseudochromosomes was obtained from GOAT,  which estimates it based on comparative karyotype data from ancestral taxa (as indicated in the main text) rather than direct cytogenetic observation for this species. We have now modified the text to clarify this point better. In contrast, our assembly provides species specific evidence, revealing 40 pairs of autosomes plus two sex chromosomes. In addition, 11 scaffolds remain unplaced in the current assembly. 
2. As noted in our response to the other reviewer, a full genome annotation for this species is not yet available: it is currently being generated and will be released as soon as completed. 
3. We thank the reviewer for pointing this out. As suggested we have now included the genome size estimation method - GenomeScope2 with a k-mer length of 21. In addition, we have also included the corresponding software versions BUSCO v5.5.0 and Merqury v1.3, as also suggested by the other reviewer. Furthermore, we now report the number and kind of interventions made during manual curation. 
4. We have modified the highlighted examples to improve clarity and formality.
5.  Modified accordingly.
6. We have clarified the text, separating the RNA-seq data production from the rest of the data, highlighting that the purpose of its  production is to aid future annotation efforts. We include transcriptomic data for the purpose of efficiency. Collecting RNA from the same specimen (or from different specimens) during the same sampling event is important and saves time, effort and costs when it comes to annotation of the reference genomes produced. The annotation step is downstream of the assembly and can take substantially more time, thus we release the assemblies as soon as possible and decouple the annotation step from necessary inclusion in the Genome Note.
7.  All relevant metadata, including the collection site and coordinates are are available under the umbrella BioProject PRJEB86373 (https://www.ebi.ac.uk/ena/browser/view/PRJEB86373 ) and in the ERGA portal (https://portal.erga-biodiversity.eu/data_portal/928672). Both links are included in the main text. 

# Reviewer report: Reviewer response for version 1

**DOI:** 10.21956/openreseurope.22356.r57670

**Published:** 2025-08-28

**Authors:** Luke W Silver

**Affiliations:** 1 University of Sydney, Sydney, Australia

Reviewer Comments

 The authors generate a draft genome for the Azores Bullfinch, a IUCN Vulnerably listed species, using ONT, HiC and Illumina data. Overall the methods of genome assembly and quality checking are sound and will provide a useful resource, however, there are a few details which would improve the data note.

 Include sequencing technology used in the Abstract

 Where is the Azores archipelago located in wider context – i.e. XX km west of Portugal in the Atlantic Ocean or include a figure of the location, maybe a panel with an image of the Azores Bullfinch

 “As a result of” instead of “due to”

 “A high quality..” instead of “this kind of…”

 “Sequencing” instead of “used”

 What type of RNA short read libraries – 150bp paired end?

 How many reads were generated by RNA sequencing?

 Say which data type was used in each of the steps – i.e. scaffolding using HiC data with YaHS…

 Some details of the assembly summary analysis is needed, such as the which version of BUSCO and Merqury are used in this workflow?

 Was any repeat identification undertaken?

 Which scaffold was identified as the mitochondrial genome?

 Method details are needed for how the sex scaffolds were identified

 Were the RNA seq reads used for any analysis or just generated for ENA submission?

 If there are any other genomes for closely related species is it expected that there are 13 macrochromosomes and then microchromosomes or is the number of macrochromosomes unknown?

Are sufficient details of methods and materials provided to allow replication by others?

Partly

Is the rationale for creating the dataset(s) clearly described?

Yes

Are the datasets clearly presented in a useable and accessible format?

Yes

Are the protocols appropriate and is the work technically sound?

Yes

Reviewer Expertise:

Genome assembly, genetic diversity

I confirm that I have read this submission and believe that I have an appropriate level of expertise to confirm that it is of an acceptable scientific standard, however I have significant reservations, as outlined above.

# Response

**Published:** 2026-01-29

**Authors:** Rita Monteiro

We thank the reviewer for their comments. We have addressed all of them as following:   

1. Added to the Abstract
2. We included a new figure (Figure 1) that shows the wider spatial context in relation to the nearest continental grounds, and the location of the Azores Bullfinch range inside the São Miguel Island. We also include a photo of the Azores Bullfinch.
3. Modified accordingly.
4. Modified accordingly.
5. Modified accordingly.
6. Were produced 150bp paired end RNA libraries. This information was added to the main text.
7. 91MR were generated, added to the text.
8. We assembled contigs from ONT data with Hi-C for phasing. We scaffolded with YaHS using Hi-C data.  K-mer counts for estimating genome size, QV and k-mer completeness were performed on the combined set of Illumina WGS and filtered ONT reads. This information was added to the text.
9. For the assembly summary analysis we used BUSCO v5.5.0 and Merqury v1.3. This information was added to the main text.
10. Yes, repeats were identified using RepeatMasker (Smit, AFA, Hubley, R & Green, P., 2013-2015), (version 4.0.5 with parameters -nolow -engine "RMBlast"), dustmasker (Morgulis, A., et al., 2006), and tandem repeat finder, TRF (Benson, G., 1999). In addition to the Repbase (Bao, W., et al, 2015) library, where available, a custom repeat library is used with RepeatMasker. Custom libraries are created using RepeatModeler2 (Flynn, J.M., 2020)
11. The mitochondrial genome corresponds to chr OZ239036.1. This was added to the main text. 
12. We have added the following text to the methods:
  “The sex chromosomes were identified by sequencing coverage. Assignment of Z and W chromosomes was according to conservation of length: bPyrMur chrZ (OZ238997.1: 86793913 bp) vs. bGalGal chrZ (CM028522.1: 86044486 bp). Whole genome alignment against GRCg7b (GCA_016699485.1) confirmed these assignments.”
13. As indicated, RNA sequencing was performed for transcriptome profiling with the primary aim of supporting genome assembly and annotation. Although a full genome annotation for this species is not yet available, it is currently being generated and will be released as soon as it becomes available. 
14. We did not include any information with respect to macro- vs. microchromosomes as there is some debate in the literature as to the length threshold (35 Mb? 30? 20 Mb?). It seems that the categorization is done by karytyping and varies from one bird species to the next. For P. murina, the clearest size break occurs between about 23 and 28 Mb. It has 10 autosomes and the Z and W that are longer than 28 Mb and the rest (30 autosomes) are shorter than 23 Mb, the shortest being about 3.5 Mb.  We have added this information to the text but avoid the macro- micro- labels.

 
